# Raman spectra and defect chemical characteristics of Sr(Ti,Fe)O_3−*y*_ solid solution of bulk pellets *vs.* thin films†

**DOI:** 10.1039/d3ta04818g

**Published:** 2023-11-28

**Authors:** Eva Sediva, Jennifer L. M. Rupp

**Affiliations:** a Electrochemical Materials, Department of Materials Science and Engineering, Massachusetts Institute of Technology 77 Massachusetts Av. MA 02139 USA jrupp@mit.edu; b Electrochemical Materials, Department of Electrical Engineering and Computer Science, Massachusetts Institute of Technology 77 Massachusetts Av. Cambridge MA 02139 USA; c Department of Chemistry, Technical University of Munich 85748 Garching Germany

## Abstract

Sr(Ti,Fe)O_3−*y*_ perovskite solid solutions are relevant functional materials for energy conversion and electronic devices such as solid oxide fuel and photoelectrochemical cells, electrolyzers, oxygen sensors, resistive random access memories or synaptic transistors. The Raman spectra and vibrational characteristics of the Sr(Ti,Fe)O_3−*y*_ materials class are suitable for describing their defect chemistry and the iron valence state, which governs a multitude of its mixed ionic–electronic transport and other characteristics. We synthesize a standard range of compositions containing 1–75 mol% of iron including the end members in the form of macrocrystalline bulk pellets, nanocrystalline poly- and single crystalline thin films. Through the change in both iron substitution level and microstructure, we directly see the effect of defect chemistry such as its phase, transition metal ion valence and oxygen nonstoichiometry on the Raman spectra. These are discussed in terms of *in* and *ex situ* experiments under oxidizing/reducing atmosphere. In contrast to long range structural X-ray diffraction measurements, Raman spectroscopy provides valuable insights into oxygen vacancy ordering and oxygen nonstoichiometry for the Sr(Ti,Fe)O_3−*y*_ material class.

## Abstract 2

We measure and interpret Raman spectra of Sr(Ti,Fe)O_3−*y*_ solid solutions from a defect chemical perspective. We compare single- and polycrystalline thin films as well as bulk pellets as a function of iron content.

## Graphical abstract

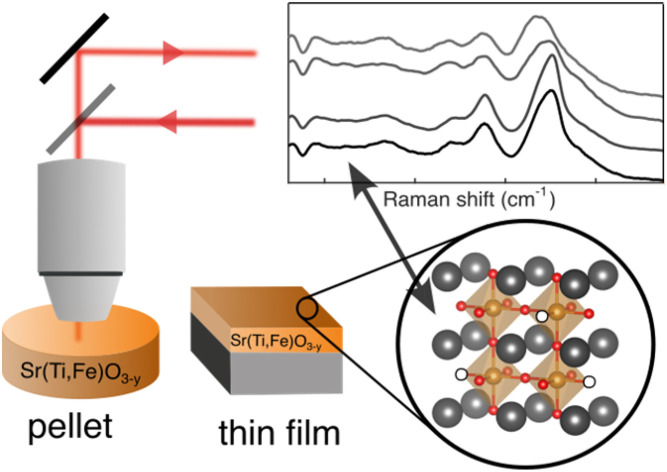

## 1 Introduction

**Fig. 1:**
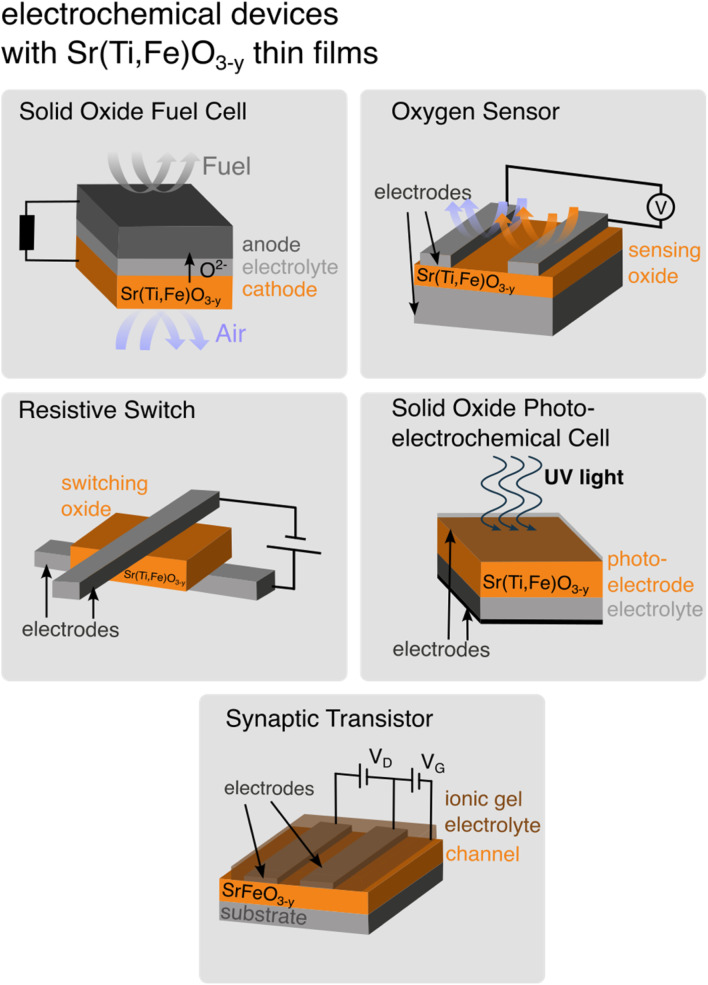
Electrochemical devices integrating Sr(Ti,Fe)O_3−*y*_ thin films.

Solid solutions of the perovskite family Sr(Ti,Fe)O_3−*y*_ are important due to their ability to vary their physical and chemical properties with composition, iron valence state, and oxygen nonstoichiometry. These can determine the material's transport,^1^ electronic,^2^ magnetic^3,4^ and optical^5,6^ properties, crucial for applications in electrochemical devices,^7^ such as electrodes for solid oxide fuel^8–10^ and photoelectrochemical cells,^11,12^ oxygen sensors,^13,14^ synaptic transistors^15,16^ or memristive devices,^17–19^Fig. 1 and review in ref. 20. Such devices are often fabricated in thin film form exhibiting conductivities,^21,22^ optical transmittance^23,24^ or point defect chemistry^25^ dependent on the processing and the nanostructure characteristics, which differ from their bulk pellet, tape or single crystalline counterparts. Easy, non-destructive and *in situ* thin film characterization techniques are essential for controlling the device fabrication and engineering their performance. Raman is a materials characterization tool measuring normal vibrational modes through inelastic scattering of an excitation laser. The strength of the technique is its sensitivity to small and local atomic displacements, that inevitably occur with defect chemical changes. In this work we aim to showcase the abilities of Raman to describe oxygen defect chemistry on the model of Sr(Ti,Fe)O_3−*y*_ solid solutions. We interpret the Raman spectra of Sr(Ti,Fe)O_3−*y*_ solid solutions from their defect-chemical perspective in order to highlight Raman as a fast, nondestructive but yet precise materials characterization technique for the broader community of ceramists and electrochemists working with this material class.

### 1.1 Physical and chemical properties of Sr(Ti,Fe)O_3−*y*_ solid solutions and its end members SrTiO_3_ and SrFeO_3−*y*_

It is illustrative to start the discussion of the materials class with the end members of the solid solution, SrTiO_3_ and SrFeO_3−*y*_, in order to later understand the properties of the intermediate compositions as an alloy of their end members.^26,27^

**Fig. 2:**
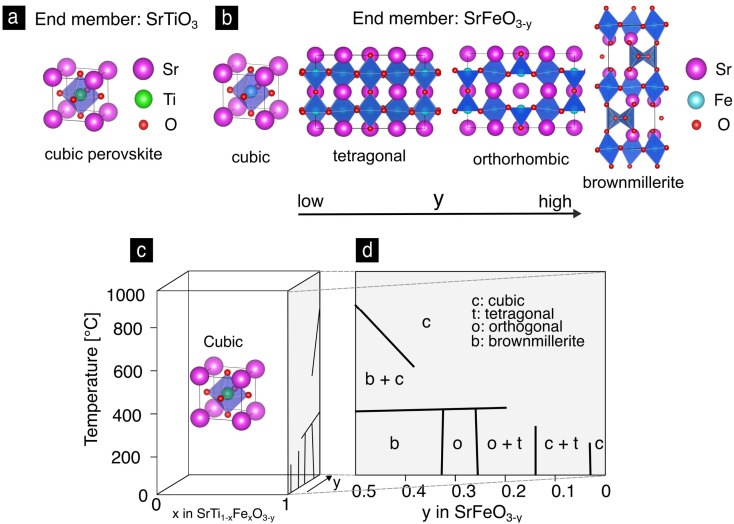
(a) The perovskite cubic lattice unit cell of SrTiO_3_. (b) Unit cells of the phases of SrFeO_3−*y*_ in the order of decreasing oxygen content. (c) Phase diagram of the Sr(Ti,Fe)O_3−*y*_ solid solution as a function of temperature, composition and nonstoichiometry. The iron oxide side of the phase diagram with the different phases is highlighted for clarity,^46^ (d).

Strontium titanate, SrTiO_3_, is a wide band gap oxide (3.2 eV) and is among the most studied electroceramics.^28^ SrTiO_3_ has been investigated in terms of its oxide charge transport properties in the bulk and grain boundaries,^29–31^ defect chemistry^32–34^ or oxygen exchange.^35^ The cubic perovskite unit cell, Fig. 2a of SrTiO_3_ is stable in both reducing and oxidizing atmospheres and over a wide range of temperatures from 105 K^36^ up to its melting point of 2353 K.^37^ In contrast, strontium iron oxide, SrFeO_3−*y*_, has a rich phase diagram due to structural accommodation of oxygen vacancies as a result of the mixed valence state of the iron ion. This has been recently exploited to control its topotactic phase transitions through electrochemical gating^38^ resulting in applications in neuromorphic,^15,16^ electrochromic^39,40^ or magneto-ionic^4^ devices. In Fig. 2b the crystal unit cells of the SrFeO_3−*y*_ phases are depicted in the order of decreasing oxidation. At high oxidation levels SrFeO_3_ grows in the cubic perovskite structure analogous to SrTiO_3_.^41^ The symmetry lowers from cubic to tetragonal, orthorhombic and finally to the brownmillerite phase through the progressive reduction of Fe^4+^ by Fe^3+^ in the crystal lattice.^42–45^ The phase diagram of SrFeO_3−*y*_ and its relation to the Sr(Ti,Fe)O_3−*y*_ solid solutions is presented in Fig. 2c, as reported by Mizusaki *et al.*, Fig. 2d.^46^

Next we describe the structural properties of the intermediate compositions of the Sr(Fe,Ti)O_3−*y*_ solid solutions, which can be described as a mixture of strontium titanate, SrTiO_3_, and strontium ferrite, SrFeO_2.5_.^2,26,27,47^ The solid solution is formed without phase separation or oxygen vacancy ordering, Fig. 2c, which has been confirmed by X-ray diffraction (XRD)^48–51^ and neutron scattering experiments.^52^ The cubic phase is formed in compositions reaching up to 90 mol% of iron.^50,53^ However, transmission electron microscopy (TEM) and X-ray absorption spectroscopy (XAS) revealed local unit cell superstructures^54^ and Fe^3+^ in a tetrahedral environment^55^ in compositions above 50 mol% Fe. Generally, the cubic perovskite phase is stabilized with increasing titanium contents^2,56^ and temperature.^57^

The description of the Sr(Fe,Ti)O_3−*y*_ solid solutions from a defect chemical and electronic perspective strongly depends on the content of the mixed-valent iron cation. Compositions with less than 1 mol% iron can be considered dilute solid solutions, and can be described as an acceptor doped wide band gap electroceramic with predominantly electronic conductivity.^27,58^ As the concentration of iron increases above the dilute solution limit (*ca.* 1 mol%) the nature of the iron electronic states becomes extended. The Fe^3+^/Fe^4+^ states lie on top of the valence band, and form covalent admixtures with the O 2p states. The width of this iron band increases with iron content as the iron states overlap with each other and with O 2p states.^2^ Consequently, the valence band shifts closer to the conduction band with increasing iron content lowering the Sr(Fe,Ti)O_3−*y*_ band gap up to *ca.* 1.9 eV in SrFeO_2.5_. Concurrently, the ionic and electronic conductivity increase turning the material into a mixed ionic–electronic conductor.^2,9,58,59^ The oxygen nonstoichiometry generally depends on temperature, oxygen pressure and iron concentration.^35^ This means that the transport and electronic properties of Sr(Ti,Fe)O_3−*y*_ solid solutions can be controlled by their composition, environment or voltage, which makes them attractive for many applications.

Lately the thin film form of the Sr(Ti,Fe)O_3−*y*_ solid solutions is increasingly requested for integration in various electrochemical devices^8,18,60,61^ and for model electrochemical experiments, which take advantage of the well defined surfaces and geometries of thin films.^13,62^ In general, thin films have different properties than bulk materials.^22,63,64^ In polycrystalline films this is a result of an increased grain boundary *vs.* grain volume due to a reduced average grain size and in single crystalline films it is an absence of grain boundaries. Other effects such as cation nonstoichiometry or dislocation density can create additional differences between thin films and bulk materials. For example, a difference in the electronic properties between bulk and thin film structures was found in films doped up to 5 mol% Fe, which have shown evidence of containing the Fe^2+^/Fe^3+^ pair of oxidation states rather than the Fe^3+^/Fe^4+^ combination known from bulk pellets.^25,65^ Also, conductivities of thin films vary from their bulk pellet counterparts. Namely, in 0.37 mol% Fe-doped SrTiO_3_ thin films the conductivity was three orders of magnitude smaller than in the bulk pellet^66^ and in Sr(Ti,Fe)O_3−*y*_ thin films with 35 mol% Fe the difference was one order of magnitude.^14^ The differences in defect chemistry between bulk and thin films can arise from the non-equilibrium states attained through film deposition techniques, substrate induced strains or space charge effects occurring for nanocrystalline grain sizes, which are close to the Debye length of the material. The different defect states induced by the thin film growth can have an effect on the bond lengths and structural distortions. Traditionally structural properties of Sr(Ti,Fe)O_3−*y*_ thin films are investigated by electron microscopy and X-ray techniques. These include TEM and XAS, which however require large-scale equipment or facilities. Raman spectroscopy is a lab-scale, thin film-suitable technique that also provides information on short range atomic ordering. We present here a characterization and spectral interpretation of Sr(Ti,Fe)O_3−*y*_ thin films and bulk pellets to serve as a reference for the broader community of ceramists or electrochemists working with these materials.

### 1.2 Raman spectroscopic characteristics of the Sr(Ti,Fe)O_3−*y*_ solid solutions and their end members

In general, the investigations of structure, phase, and defect chemistry of Sr(Ti,Fe)O_3−*y*_ solid solutions are essential for their integration into any electrochemical devices. Usually, the material's phase is confirmed by XRD, which is an averaging technique providing no information about local atomic arrangements. Raman spectroscopy on the other hand can give useful information on local symmetry breaks caused for example by oxygen vacancy ordering or iron valence state and can therefore serve as a strong complementary technique for structural and defect chemical investigations.

Generally in the perovskite lattice Raman can probe the oxygen octahedra tilts, rotations or deformations, that can exclusively activate certain phonon modes.^67,68^ Sr(Ti,Fe)O_3−*y*_ solid solutions were investigated with Raman spectroscopy in the form of ceramic powders^69^ and nanoparticles,^70^ single crystals^71,72^ and polycrystalline thin films.^62,73^ Importantly, we have previously shown that the oxygen nonstoichiometry in Sr(Ti,Fe)O_3−*y*_ thin films with can be quantified by the frequency of its oxygen vibration.^73^ However, Sr(Ti,Fe)O_3−*y*_ spectra of the bulk pellets and thin films including epitaxially grown thin films have not been systematically compared. Since the defect chemistry between the bulk and thin films can substantially vary^14,65,66^ such a comparative study can serve as a basis for thin film characterization and *in situ* electrochemical device measurements.

In this paper we synthesize and investigate Sr(Ti,Fe)O_3−*y*_ solid solution system to describe its Raman characteristics over the whole composition range and for various microstructures ranging from nanocrystalline thin films to macrocrystalline bulk pellets, also including single crystalline thin films for a grain boundary free model material case. First, we present and interpret the Raman spectra of the SrTiO_3_ and SrFeO_3−*y*_ end members. Second, we discuss the Raman spectra of the full Sr(Ti,Fe)O_3−*y*_ solid solutions in the range of 1–75 mol% Fe comparing effects on Raman vibrational characteristics of random and ordered oxygen vacancies, and iron valence state complemented also by *in situ* electrochemical reduction experiments. The concepts developed here ultimately contribute to the understanding of the influence of defect chemistry on the vibrational modes in the Sr(Ti,Fe)O_3−*y*_ solid solution, which enables characterization of materials physical and chemical properties based on defect chemistry described crucial for the future design of functional electrode and electrolyte materials in energy and information devices.

## 2 Experimental

SrTi_1−*x*_Fe_*x*_O_3−*y*_ bulk pellet and thin film samples with compositions of *x* = 0, 0.01, 0.25, 0.3, 0.5, 0.75, 1 were considered in this study. The bulk pellets were prepared from Sr(Ti,Fe)O_3−*y*_ powders by uniaxial and isostatic pressing and their phase was confirmed by X-ray diffraction. The relevant powders were produced by a conventional solid state synthesis by ball milling the appropriate amounts of SrCO_3_, TiO_2_ and Fe_2_O_3_ and sintering powders at 1350 °C for 5 hours. Thin film samples were deposited by pulsed laser deposition (Surface, Germany) with a KrF 248 nm excimer laser. The deposition was performed at 650 °C under 0.027 mbar oxygen pressure for 2000 shots with a laser fluence of 1.9 J cm^−2^. The distance of the oxide target and the substrate was 8 cm. The film thickness was about 100 nm. The substrates for the oxide thin films were randomly oriented sapphire substrates (Stettler Sapphire, CH) with 80 nm thick bottom layer of e-beam evaporated platinum (Plassys MEB 550, France). The platinum bottom layer serves to absorb the Raman signal and prevent the sapphire substrate Raman peaks to appear in the final spectrum.

Additionally, oriented thin films (SrTi_0.99_Fe_0.01_O_3−*δ*_ and SrTi_0.7_Fe_0.3_O_3−*y*_) were grown on (100) LaAlO_3_ substrates (CrysTec, Germany) with pulsed laser deposition system. The substrate temperature was 650 °C, 0.027 mbar, the laser pulse frequency 2 Hz, the substrate–target distance was 8 cm, laser fluence 1.9 J cm^−2^ and the number of shots was 2000.

The XRD of the pellets was measured on the PANalytical X'Pert Pro MPD with the Cu K_α_ wavelength and the polycrystalline thin films on the PANalytical X'Pert3 MRD with the Cu K_α_ wavelength in grazing incidence with 0.4° angle. Both scans were measured in the scan range of 2*θ* = 20–140°. The XRD patterns of the oriented thin films grown on LaAlO_3_ were measured on the Bruker D8 High-Resolution XRD with the Cu K_α_ wavelength in the scan range 2*θ* = 20–60°. The indexing and refinement were performed with the PANalytical X'Pert HighScore Plus software.

Raman spectra were taken with a confocal WITec alpha300 R Raman microscope (WITec, Germany) with three different excitation wavelengths of 457 nm (2.71 eV), 532 nm (2.33 eV) and 633 nm (1.96 eV) and a grating of 1800 grooves per mm. For laser focusing a 100× objective with a numerical aperture (NA) of 0.9 Zeiss microscope was used, which gives an approximate laser spot size of 0.8 μm for the 633 nm laser. The laser energy was adjusted according to the sample between 20 μW and 5 mW. Several different integration times were used according to the specific samples. To minimize the resonant contributions to the spectra, only spectra of the 633 nm excitation wavelength are presented if not stated otherwise.

The *in situ* oxidation experiments were performed in the HFS600 Linkam (Resultec, Germany) stage using pure oxygen or argon (50 sccm). Here a high working distance (50×, NA = 0.6) objective (Zeiss, Germany) was used to focus on the sample to accommodate for the stage height.

## 3 Results and discussion

### 3.1 End members of the solid solution: SrTiO_3_ and SrFeO_3−*y*_

**Fig. 3:**
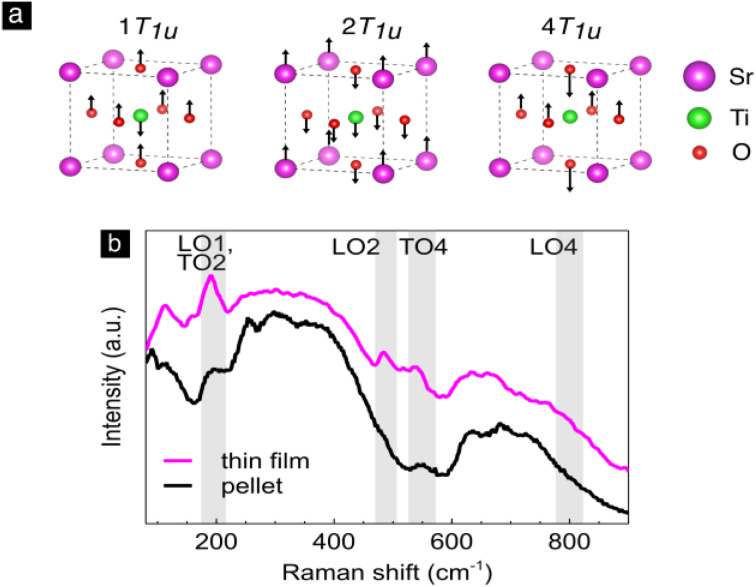
Strontium titanate, SrTiO_3_: (a) the three triply degenerate first-order IR active vibrational modes depicted in the *z* direction of the unit cell. (b) The Raman spectrum of SrTiO_3_ thin film and bulk pellet.

Strontium titanate, SrTiO_3_, has a cubic crystal structure that has no allowed first-order Raman modes since all atoms sit on a center of inversion. From group theoretical analysis it follows that SrTiO_3_ has at the *Γ* point of the Brillouin zone four T_1u_ and one silent T_2u_ mode. Three of the T_1u_ modes are infrared active and one is acoustic.^74^ In Fig. 3a the T_1u_ first-order lattice vibrational modes in the *z* direction of the cell are depicted. The first mode, 1T_1u_, in Fig. 3a corresponds to the Ti–O–Ti bending and is the soft mode connected to the ferroelectric instability.^75,76^ The second mode corresponds to the translation of Sr against the TiO_6_ octahedra and the last to Ti–O stretching.

In Fig. 3b the comparison of Raman spectra of the SrTiO_3_ thin film and the bulk pellet at room temperature is shown. Both spectra clearly show pronounced broad Raman features from 200 to 450 cm^−1^ and from 600 to 800 cm^−1^. These we assign to second-order Raman scattering in SrTiO_3_.^75,77^ Importantly, the material can reveal also first-order modes when processed as a thin film, which was confirmed in single crystalline^78,79^ as well as polycrystalline^80^ thin films. This is possible due to the breaking of translational and/or inversion symmetry from intrinsic defects or oxygen nonstoichiometry,^80^ impurities^81^ or strain^78,79^ often present in thin films. Turning to the thin film spectrum, Fig. 3b, modes at 180, 486 and 539 cm^−1^ are visibly strengthened with respect to the bulk pellet spectrum. We assign these peaks to the infrared active T_1u_ symmetry modes and accordingly to their transverse and longitudinal components: second transverse optical (TO2) and first longitudinal optical mode (LO1), second longitudinal (LO2) and fourth transverse optical mode (TO4).^82,83^ The large frequency splitting of the Raman vibrational modes into the transverse and longitudinal components is a characteristic of perovskites such as SrTiO_3_, BaTiO_3_ (ref. 84) or PbTiO_3_ (ref. 85) see ref. 86 for details.

**Fig. 4:**
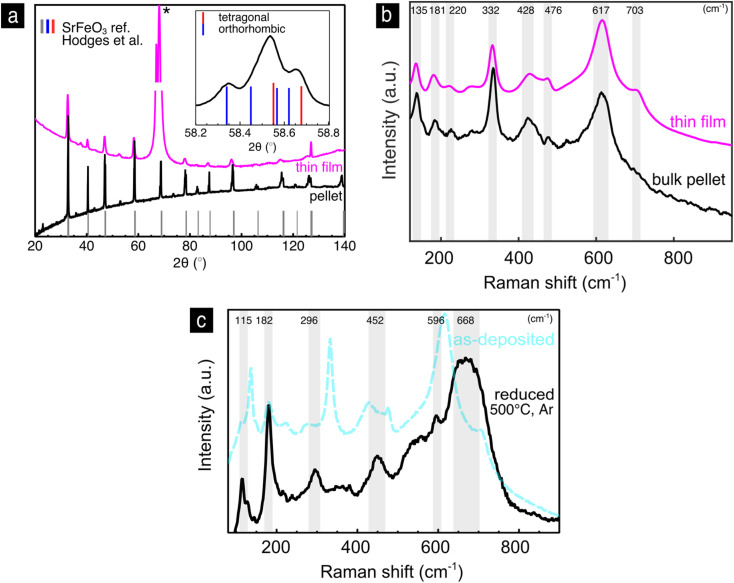
Strontium iron oxide, SrFeO_3−*y*_: (a) XRD-pattern of the thin film and bulk pellet with an inset of a sample reflection from the bulk pellet with a reference of the tetragonal and orthorhombic phases. (b) Raman spectra of the SrFeO_3−*y*_ thin film and bulk pellet. (c) Comparison of the Raman spectra of the as-deposited and reduced thin films.

We now turn to the strontium iron oxide, SrFeO_3−*y*_, being the second end member of the solid solution series. Looking first to the thin film XRD pattern, Fig. 4a, we find major diffraction peaks at 2*θ* = 32.6, 40.3, 46.9, 58.3 and 126.9°, which correspond to the ref. 43 of cubic SrFeO_3_ (grey in Fig. 4a). (The strong diffraction peak at 66° marked with (*) belongs to the single crystalline sapphire substrate.) The bulk pellet pattern, Fig. 4a, has the same reflections as the thin film pattern, however, noticeable are also peak splittings and intermediate reflections. A peak splitting example is shown in the inset of Fig. 4a, where reflections corresponding both to the tetragonal and orthorhombic phases of SrFeO_3−*y*_ are present. Therefore, we conclude, that the phase of the SrFeO_3−*y*_ bulk pellet is a mixture of the orthorhombic and tetragonal phases, which means that the oxygen nonstoichiometry *y* in SrFeO_3−*y*_ is somewhere between 0.15 and 0.35 according to the phase diagram in Fig. 2c.^46^

In Fig. 4b, the Raman spectrum of the SrFeO_3−*y*_ as-deposited thin film is compared to the bulk pellet. The spectra are qualitatively similar confirming the transfer of the same phase during the pulsed laser deposition from pellet to thin film. This contrasts the XRD results, which predicted a cubic phase of the thin film. XRD in this case lacks the sensitivity to resolve the superstructure reflections in the thin film. This result highlights the sensitivity of Raman to local atomic arrangements, that can stay unresolved with XRD and shows the suitability of this method for the characterization of the SrFeO_3−*y*_ reduction extent, critical for its respective electrochemical devices.

The spectra of the SrFeO_3−*y*_ as-deposited thin film, Fig. 4b, has Raman modes at 135, 181, 220, 332, 428, 476, 617 and 703 cm^−1^. Apart from the weaker 476 and 703 cm^−1^ Raman modes, the spectrum is in agreement with experimental spectra of the orthorhombic–tetragonal phase mixture of SrFeO_3−*y*_ measured by Radheep *et al.*^87^ and of spectra measured with *y* = 0.31 by Adler *et al.*^88^ and with *y* = 0.39 by Barkalov *et al.*^89^ The assignment of the weak modes at 476 cm^−1^ and 703 cm^−1^ is unclear, however, they could belong to the orthorhombic SrFeO_3−*y*_ phase, since similar weak modes were reported by Damljanovic.^42^ Table 1 in the ESI† summarizes the symmetry allowed Raman modes for the tetragonal (*I*4/*mmm*), orthorhombic (*Cmmm*)^42^ and the brownmillerite (*Imma*)^90,91^ phases. The tetragonal phase has 31 allowed Raman active modes, the orthorhombic phase 21 and the brownmillerite phase 51. It is clear that not all modes appear in the spectrum, and we cannot unambiguously assign the phase based solely on the symmetry analysis.

We further reduce the as deposited SrFeO_3−*y*_ thin film at 500 °C in argon to probe the effect of the different redox states of iron on the phase of SrFeO_3−*y*_. After reduction the sample is quenched (150 °C min^−1^) to room temperature where the Raman spectrum is measured, Fig. 4c. The spectrum shows peaks at 115, 182, 296, 452, 596 and 668 cm^−1^. The SrFeO_2.5_ brownmillerite phase Raman spectrum has a distinctive enhanced mode around 660 cm^−1^,^89,91^ which has been attributed to the symmetrical stretching of oxygen around the Fe^3+^ transition metal ion.^91^ Additional Raman modes of the SrFeO_2.5_ brownmillerite phase were found around 290, 440 and 600 cm^−1^.^89,91^ For the complete assignment of their symmetry, polarized Raman on single crystalline samples would be necessary. In conclusion, the SrFeO_3−*y*_ thin films reveal for the as-deposited state a mixture of tetragonal and orthorhombic phases, which changes to the brownmillerite phase upon reduction at 500 °C in argon.

### 3.2 Phase and Raman spectra of the Sr(Ti,Fe)O_3−*y*_ solid solutions

**Fig. 5:**
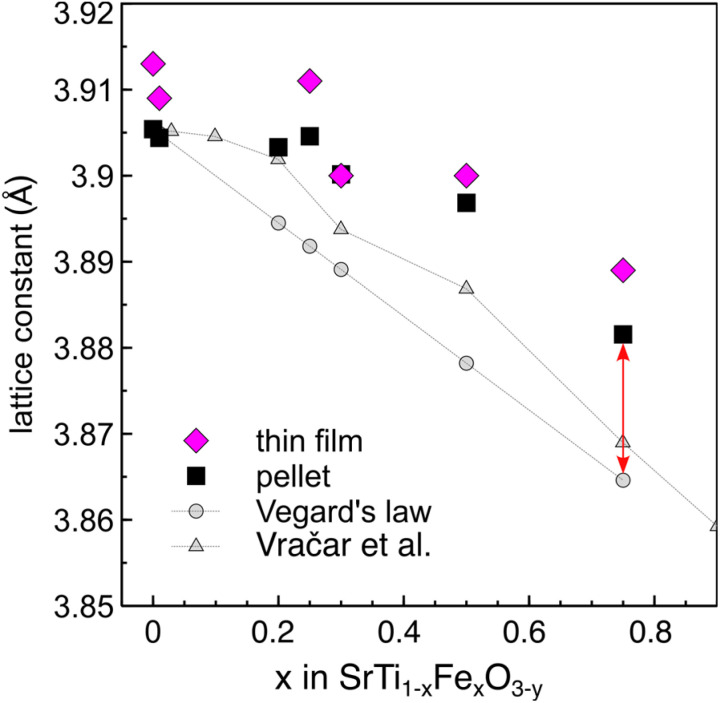
X-Ray diffraction. Refined lattice constants from the XRD patterns of Sr(Ti,Fe)O_3−*y*_ thin films and pellets as a function of the iron content. For comparison, we show Vegard's law prediction based on oxidized Sr(Ti,Fe)O_3_ solid solutions and the results of powders oxidized under 600 bar O_2_ from Vračar *et al.*^43,46,69^

The XRD patterns of the Sr(Ti,Fe)O_3−*y*_ solid solutions, Fig. S1,† show the reflections of the cubic perovskite phase for all the considered compositions. The lattice constants were determined through a standard refinement process of the perovskite cubic phase. The empirical Vegard's law describes the change in lattice parameter of a solid solution as the weighted mean two fully intermixable phases (the two end members of the solid solution SrTiO_3_ and SrFeO_3−*y*_). The iron ion changes its size depending on its valence and their respective amounts then determine the average lattice parameter. We plot a reference Vegard's law in Fig. 4 with the assumption that all iron has the 4+ valence. In Fig. 5 we compare the measured lattice constants to the Vegard's law of oxidized Sr(Ti,Fe)O_3_ (only Fe^4+^ present) as well as to the lattice constants measured by Vračar *et al.* of powders annealed under an oxygen pressure of 600 bar.^69^ The lattice constants of both thin films and pellets decrease with increasing iron content, are however much larger than Vegard's law predicts for complete oxidation (red arrow in Fig. 5). This points to the substitution of Ti by both Fe^4+^ with a smaller and Fe^3+^ with a larger ionic radius than Ti^4+^.^58,59,69^ The slightly larger lattice constants of Vračar *et al.*^69^ as compared to the oxidized prediction from Vegard's law show that full oxidation is not achieved even when annealing under 600 bar O_2_.^92^ In summary, the refinement of the XRD patterns show that all the samples contain a mixture of Fe^4+^ and Fe^3+^ valence states, however, it is not possible to quantify their respective content.

**Fig. 6:**
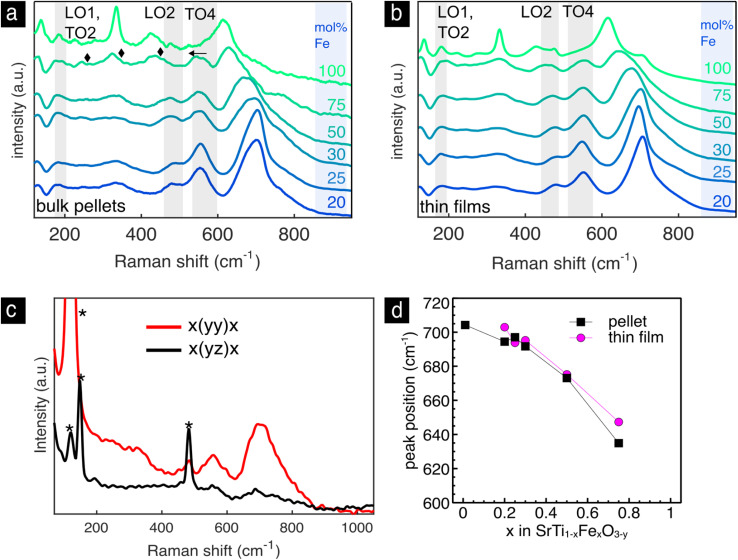
Sr(Ti,Fe)O_3−*y*_ solid solutions. Raman spectra of the bulk pellets (a) and thin films (b) with the iron content indicated next to each spectrum. Highlighted are the first order IR-active T_1u_ modes of the cubic perovskite lattice. In the SrTi_0.25_Fe_0.75_O_3−*y*_ bulk pellet we highlight the new modes with diamond shapes and the red shift of the TO4 mode with an arrow. (c) Polarized Raman spectra of an oriented thin film with 30 mol% Fe grown on LaAlO_3_ substrate. Raman modes denoted with (*) belong to the LaAlO_3_ substrate. (d). Peak positions of the enhanced oxygen stretching mode as a function of the composition for the bulk pellets and thin films from (a) and (b).

The Raman spectra of the bulk pellets and thin films of the Sr(Ti,Fe)O_3−*y*_ solid solutions are shown with respect to the iron concentration in Fig. 6a and b. In both pellets and thin films, Fig. 6a and b, we find the T_1u_ modes (LO1, TO2, LO2, TO4) of the cubic lattice analogous to SrTiO_3_, Fig. 3b, and an additional highly enhanced mode around 700 cm^−1^. The T_1u_ modes are broadened and strengthened with respect to pure SrTiO_3_, which can be attributed to the B-site disorder breaking the translational and inversion symmetry. In the bulk pellet with 75% iron we see three extra modes appear at 245, 324 and 435 cm^−1^. Additionally, the LO2 mode disappears and the TO4 mode red-shifts with respect to the other compositions. The changes mimic the SrFeO_3−*y*_ spectrum in the tetragonal–orthorhomic phase mixture, and we therefore assign their presence to an oxygen vacancy ordering.^54^ This is in accordance to previous literature results, which predict a higher degree of structural distortions with iron concentrations above 50 mol% Fe.^54,55^ Interestingly, these spectral changes are not present in the thin film of the same iron concentration, which preserves the cubic phase by randomly distributing the oxygen vacancies. Raman spectroscopy measures normal vibration modes, whose number and position change upon symmetry breaks or changes in the material such as their redox state. This ability is highlighted in the non-destructive identification of oxygen vacancy ordering in the SrFeO_3−*y*_ pellet and is accessible *via* Raman spectroscopy. On the other hand, XRD is an averaging technique that measure mean atomic positions. For these reasons and in this case, it is impossible *via* XRD to clearly show the evident oxygen vacancy ordering in the pellet sample of SrFeO_3−*y*_.

Next, we will discuss the intense mode around 700 cm^−1^, which is visible in all spectra in Fig. 6. It is connected to an oxygen vibration around Fe^4+^,^73^ which has been assigned in prior work through *in situ* electrochemical titration, see ref. 73 for further details. Similar enhanced modes are commonly measured and characterized in other B site substituted perovskite solid solutions.^93–98^ They have been assigned to a charge transfer activated oxygen breathing,^93,96,99^ ordering of the transition metal cations on the B site,^98,100^ and a Jahn–Teller distortion.^69^ We do not expect the band to be related to B site ordering as no evidence of this has been found so far in current or previous studies of Sr(Ti,Fe)O_3−*y*_.^2^ A charge transfer process would be possible between two neighboring Fe atoms but unlikely between Fe and Ti. And finally, a local Jahn–Teller distortion around Fe^4+^, previously suggested to activate the enhanced mode,^69,101^ diminishes as the iron band broadens merging the d-electron energy levels.^102^ We conclude therefore, that the mode is clearly connected to the oxidized iron state but the physical origin of it remains unclear.

To assign its symmetry, we have investigated oriented SrTi_0.7_Fe_0.3_O_3−*y*_ films grown on (100) LaAlO_3_ substrates, Fig. 6d. The XRD patterns in Fig. S2† show the epitaxial nature of the film. The Raman spectra were measured in the backscattering configuration in the quasicubic *y*–*z* plane of the LaAlO_3_ substrate with both parallel and cross polarization configurations. The mode disappears in the cross polarization configuration signifying a symmetrical stretching vibration. This is in accordance to experimental^93,103^ and theoretical^101,104^ results of structurally related B site perovskite solid solutions assigning the mode to a local A_g_-like oxygen stretching mode.

### 3.3 Role of the iron valence on the Raman spectra of Sr(Ti,Fe)O_3−*y*_

The enhanced oxygen stretching mode red-shifts as the material expands during reduction and Fe^4+^ is replaced with Fe^3+^.^73^ The spectra in Fig. 6 clearly show a red-shift with increasing iron content. This apparent expansion contrasts the average lattice contraction as measured from XRD, Fig. 5. The maximum Raman frequency of the stretching mode occurs in completely oxidized samples (only Fe^4+^) around 700 cm^−1^ for all compositions.^69,73^ With the introduction of Fe^3+^ the mode down shifts in frequency. In both thin films and pellets this down shift is greater with increasing iron concentration. This has to do with the expansion of the local environment around Fe^4+^ as more Fe^3+^ neighbors are introduced. It is also evident from the difference in the measured lattice constant from the theoretical lattice constant at complete oxidation (red arrow in Fig. 5), which increases with iron content. In prior work, the oxygen nonstoichiometry has been calibrated to the position of the oxygen stretching mode for the compositions of 30 and 50 mol% Fe.^73^ In order to calculate the oxygen nonstoichiometry in the present samples we use the previously measured Raman frequency of completely reduced Sr(Ti,Fe)O_3−*y*_ (685 and 664 cm^−1^ for 30 and 50 mol% Fe), and the relative change in wavenumber (*ν*) with respect to the oxygen nonstoichiometry (*y*) 
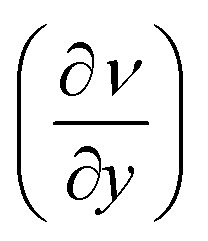
 (123 and 180 cm^−1^ for 30 and 50 mol% Fe). The resulting nonstoichiometry for the bulk pellets is *y* = 0.02, 0.12, and for the as-deposited thin films *y* = 0.074, 0.16, corresponding to 30 and 50 mol% Fe respectively. The thin films are therefore slightly more reduced than the pellets, which is manifested also in their higher oxygen vibrational frequencies, Fig. 6c. We want to stress that for an absolute and non-comparative quantification of the oxygen nonstoichiometry, a reference sample with a known composition is required. In summary, the frequency of the oxygen stretching mode gives a convenient measure of the sample's oxidation extent, and it can be used to determine the oxygen stoichiometry also quantitatively,^73^ unlike classical XRD.

**Fig. 7:**
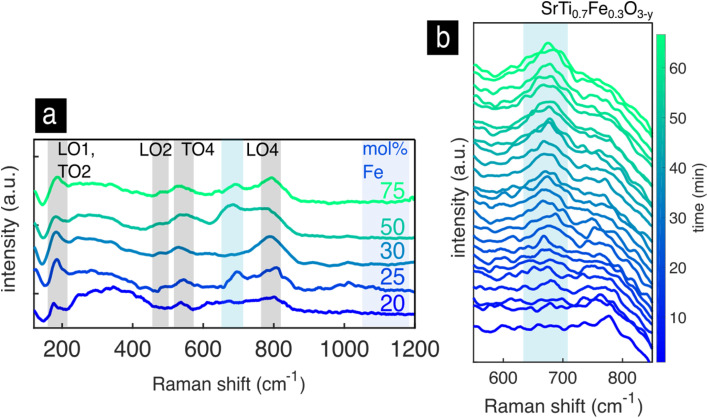
High temperature treatments of the Sr(Ti,Fe)O_3−*y*_ thin films. (a) Raman spectra of the thin film samples reduced at 500 °C under a vacuum atmosphere at 10^−6^ mbar. (b) Raman spectra of the SrTi_0.7_Fe_0.3_O_3−*y*_ thin film during an *in situ* oxidation at 450 °C starting from a thin film reduced in an argon atmosphere at 500 °C. The oxygen stretching mode is indicated in blue.

To further clarify the role of iron valence on the Sr(Ti,Fe)O_3−*y*_ Raman spectra we measure the thin films under varying atmosphere in *ex* and *in situ* experiments, Fig. 7. After reduction at 500 °C under 10^−6^ mbar vacuum the enhanced oxygen stretching mode either disappears or its intensity significantly decreases consistent with previous reports,^69^Fig. 7a. This phenomenon was explained by Blokhin *et al.*, who showed with *ab initio* calculations that the phonon density of states in the region from 620 to 760 cm^−1^ is zero when 
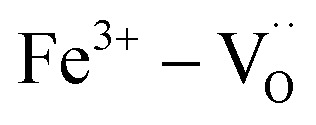
 complexes are present in the material.^104^ The absence of additional Raman modes beyond the T_1u_ cubic modes signifies that the thin films remain in the cubic phase even upon strong reduction.

To understand the potential of Raman to measure oxygen incorporation kinetics we perform an *in situ* experiment with the SrTi_0.7_Fe_0.3_O_3−*y*_ thin film, Fig. 7b. First, we reduce the sample under argon at 500 °C until the oxygen stretching mode disappears. Next, we measure spectra at 450 °C under an oxygen atmosphere, and monitor the growth of the oxygen stretching mode, signifying the ongoing oxidation of Fe^3+^ to Fe^4+^. With enhanced temperatures, Raman spectra are noisier, the intensity lowers and the peaks broaden due to increased thermal vibrations. This in turn increases the necessary acquisition time per spectrum lowering the time resolution of the *in situ* experiment. Time resolution of *in situ* Raman experiments in general will be given by the strength of the Raman bands (sample nature), experimental temperature and oxygen exchange kinetics. Additionally, Raman mode intensity depends on the sample, laser energy, laser focus and instrumentation. Therefore quantification of the laser intensity changes over time are less precise than peak positions. Here, we do not attempt to fully quantify oxygen exchange kinetics. The growing oxygen stretching mode is shifted to lower wavenumbers (*ca.* 675 cm^−1^), Fig. 7b. This is a consequence of a combination of chemical expansion during the reduction of the material and thermal expansion at higher temperatures. Since these expansions are strongly coupled,^105^ it is difficult to separate their contributions to the peak shift. In summary the *in situ* experiments confirm the connection of the oxygen stretching mode to the Fe^4+^ cation and also highlight the potential to measure oxidation kinetics with Raman, relevant for *operando* measurements of electrochemical devices.

#### 3.3.1 SrTi_0.99_Fe_0.01_O_3−*δ*_

**Fig. 8:**
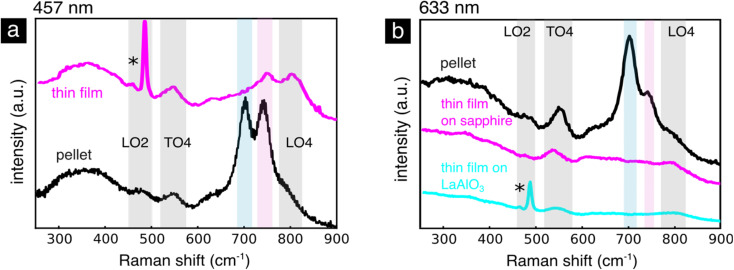
SrTi_0.99_Fe_0.01_O_3−*δ*_. (a) Raman spectra of the SrTi_0.99_Fe_0.01_O_3−*δ*_ oriented thin film compared to the bulk pellet collected using the 457 nm excitation wavelength. (b) Raman spectra of the SrTi_0.99_Fe_0.01_O_3−*δ*_ oriented and polycrystalline thin films compared to the bulk pellet collected using the 633 nm excitation wavelength. The oxygen stretching mode around Fe^4+^ is highlighted in blue and the resonantly enhanced mode in pink.

Previous reports have shown strong differences in the defect chemistry in lightly iron doped SrTiO_3_ in the bulk and thin film form.^25,65,66^ To investigate these claims, we measure spectra of SrTiO_3_ doped with 1 mol% iron in the form of oriented and polycrystalline thin films as well as a bulk pellet, Fig. 8. The thin film and pellet samples are compared in Fig. 8a using the 457 nm and in Fig. 8b using the 633 nm excitation laser wavelength. We observe that the T_1u_ modes (TO4, LO4) are present in all spectra, confirming their cubic phase as evidenced also by XRD, Fig. S1 and S2.† In the bulk pellet we observe additional two peaks arising at 704 and 740 cm^−1^. The mode at 704 cm^−1^ we attribute to the same oxygen stretching mode around Fe^4+^ as discussed above. The mode around 740 cm^−1^ is especially pronounced when using the 457 nm (2.7 eV) excitation wavelength, Fig. 8a and b. This excitation energy is close to the charge transfer from the Fe^4+^ defect energy level into the conduction band^5,106–108^ pointing to a resonantly enhanced mode connected to Fe^4+^. The origin of this mode is not clear, but it can be attributed to a different short range order of the oxygen around the iron cation.

When comparing the bulk pellet with the thin film, Fig. 8a, we notice that the oxygen stretching mode at 704 cm^−1^ disappears in both oriented (grown on LaAlO_3_) and polycrystalline (grown on sapphire) thin films, Fig. 8b. Using a combination of X-ray absorption and photoelectron spectroscopy Koehl *et al.* reported that SrTiO_3_ thin films doped with iron up to 5 mol% contain the Fe^3+^ and Fe^2+^ oxidation states,^25^ rather than the Fe^4+^/Fe^3+^ pair present in the bulk. They argue that the non-equilibrium deposition of the thin film introduces a higher concentration of point defects or that dislocations and anti-phase boundaries introduce a higher number of oxygen vacancies. The absence of Fe^4+^ in lightly Fe doped SrTiO_3_ thin films justifies the absence of the enhanced mode at 704 cm^−1^ associated to Fe^4+^, Fig. 8a and b. When using the 457 nm excitation wavelength, Fig. 8a, however, the resonantly enhanced mode around 740 nm connected to an electronic transition from Fe^4+^ does not completely disappear in the thin film sample, Fig. 8. This could be caused by a low concentration of Fe^4+^, which have been shown to form under the irradiation of wavelengths between 390 and 485 nm.^6,109^

## 4 Conclusion

Understanding the Raman mode characteristics of the materials class Sr(Ti,Fe)O_3−*y*_ and its wide range of compositions is essential to understand and design its defect chemistry by means of oxygen nonstoichiometry, oxygen vacancy ordering and the iron valence state. A multitude of mixed ionic–electronic transport characteristics or even magnetic properties can be described through the assessment of the Raman vibrational characteristics of each solid solution composition. Despite the wide use of this materials class in energy and information devices in both bulk and thin film form, making Sr(Ti,Fe)O_3−*y*_ almost a model material case, experimental description of its Raman vibration characteristics remains still scarce.

In this work we synthesize a wide set of model Sr(Ti,Fe)O_3−*y*_ solid solution compositions and study their Raman signatures in bulk pellet (macroscrystalline), polycrystalline thin film (nanocrystalline) and epitaxial films (without grain boundaries) to effectively modulate the role of grain boundaries and grain size. In this model solid solution, the presence of Fe^3+^/Fe^4+^ redox states is balanced with changes in the oxygen nonstiochiometry to keep overall electroneutrality in the defect chemistry. Clearly, the Raman spectra recorded confirm that all here exemplified compositions of the solid solution exhibit an enhanced oxygen stretching mode around 700 cm^−1^ connected to the Fe^4+^ valence state, which may serve as a material's fingerprint. Specifically, through polarized measurements on epitaxial films we assign it to the symmetric stretching of oxygen around the Fe^4+^ cation. An exception is the lightly iron doped (1 mol%) polycrystalline and epitaxial thin films, where the Fe^4+^ valence is missing. But also oxygen vacancy ordering has been verified in the pellet with high iron content (75 mol%) established through the occurrence of Raman modes that can be attributed to the tetragonal and orthorhombic phases of SrFeO_3−*y*_. Regarding the SrTiO_3_ and SrFeO_3−*y*_ end members, we identify the usual cubic phase in SrTiO_3_ bulk pellets and thin films. In SrFeO_3−*y*_ we trigger the topotactic phase transition by high temperature reduction, which we attribute to a transition from the tetragonal–orthorhombic phase mixture to the brownmillerite phase by a combination of Raman and XRD.

Collectively, we present a comprehensive study of the Raman characteristics for the model Sr(Ti,Fe)O_3−*y*_ solid solutions and reveal the varying impact of the iron valence state on oxygen vacancies. These findings can serve as a standard for the determination of defect chemical characteristics in Sr(Ti,Fe)O_3−*y*_ systems and aid the materials design of energy and information devices integrating this materials class. Typically defect chemical characteristics are determined *via* electrochemical studies through the application of electrodes. We advocate based on the findings presented that important insights on both the transition metal ion redox state, oxygen nonstoichiometry and ordering relevant for mixed ionic–electronic transport, and other properties can be fully accessed even for thin film samples *via* Raman spectroscopy.

## Conflicts of interest

There are no conflicts to declare.

## Supplementary Material

TA-011-D3TA04818G-s001
