# Supplementary material for: Raman spectra and defect chemical characteristics of Sr(Ti,Fe)O3−y solid solution of bulk pellets vs. thin films
Source: J Mater Chem A Mater. 2023 Nov 28;11(48):26752–63. doi: 10.1039/d3ta04818g (PMC10715564; doi:10.1039/d3ta04818g)
Supplement: TA-011-D3TA04818G-s001 [file TA-011-D3TA04818G-s001.pdf]

Electronic Supplementary Material (ESI) for Journal of Materials Chemistry A.
This journal is © The Royal Society of Chemistry 2023

Supporting Information to

Raman Spectra and Defect Chemical Characteristics of Sr(Ti,Fe)O_{3-y} Solid Solution of Bulk Pellets vs. Thin Films

Eva Sediva[1,2,3] and Jennifer L.M. Rupp*[1,2,4]

1 Electrochemical Materials, Department of Materials Science and Engineering, Massachusetts Institute of Technology, 77 Massachusetts Av., MA, 02139, USA
2 Electrochemical Materials, Department of Electrical Engineering and Computer Science, Massachusetts Institute of Technology, 77 Massachusetts Av., Cambridge MA, 02139, USA
3 ETHZ Department of Materials, Hönggerbergring 64, 8093, Zürich, Switzerland
4 Department of Chemistry, Technical University of Munich, 85748 Garching, Germany

*jrupp@mit.edu

Group theoretical analysis of the different phases of $SrFeO_{3-y}$

Table 1: Mechanical representations of the different $SrFeO_{3-y}$ phases

Cubic phase	$SrFeO_3$	*Pm3m*
	Raman	none
Tetragonal phase	$Sr_8Fe_8O_{23}$	*I4/mmm*
	Raman	$7A_{1g} + 7B_{1g} + 6B_{2g} + 11E_g$
Orthorhombic phase	$Sr_4Fe_4O_{11}$	*Cmmm*
	Raman	$6A_g + 6B_{1g} + 5B_{2g} + 4B_{3g}$
Brownmillerite phase	$Sr_2Fe_2O_5$	*Imma*
	Raman	$13A_1 + 12A_2 + 12B_1 + 14B_2$

XRD patterns of the thin films and bulk pellets

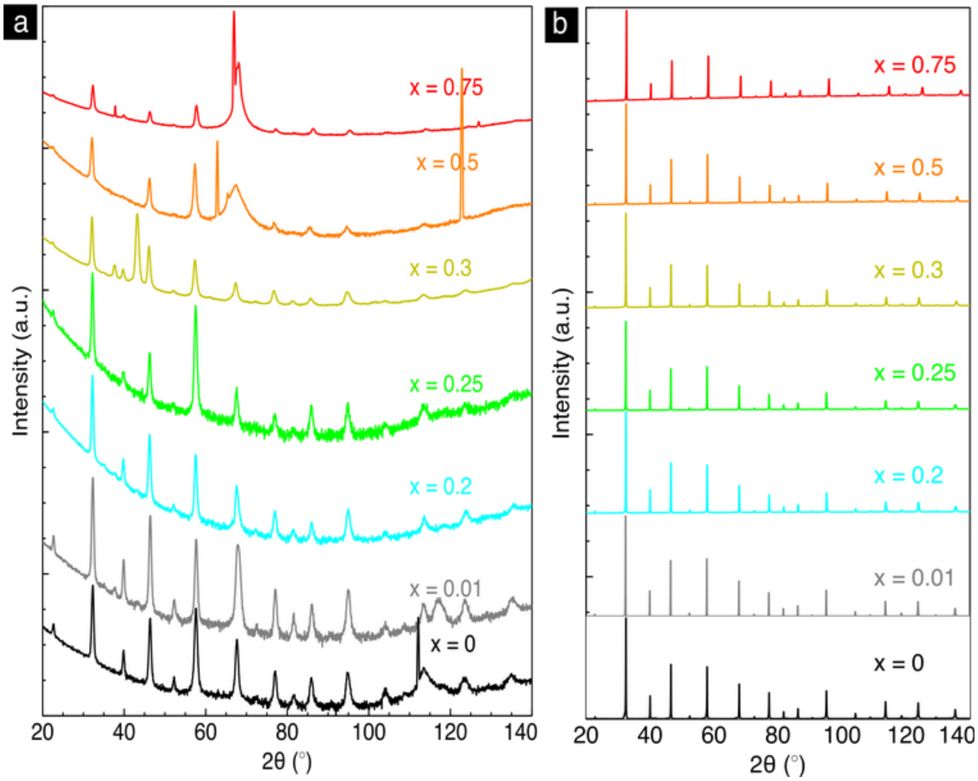

Figure S1: XRD patterns of the $SrTi_{1-x}Fe_xO_{3-y}$ thin films grown on sapphire (a) and the bulk pellets (b) for different x.

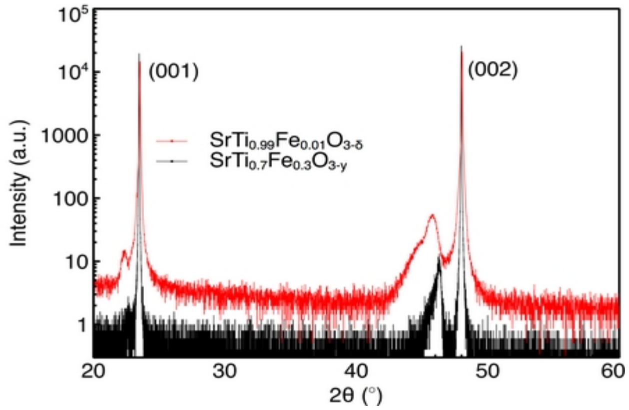

Figure S2: XRD pattern of the oriented thin films grown on $LaAlO_3$.
